# Body size of Chrysomelidae (Coleoptera, Insecta) in areas with different levels of conservation in South Brazil

**DOI:** 10.3897/zookeys.157.1083

**Published:** 2011-12-21

**Authors:** Adelita M. Linzmeier, Cibele S. Ribeiro-Costa

**Affiliations:** 1 Laboratório de Sistemática e Bioecologia de Coleoptera (Insecta), Departamento de Zoologia, Universidade Federal do Paraná, Caixa Postal 19020, 81531–980 Curitiba-PR, Brazil. Fellowships CNPq.

**Keywords:** Abundance, biodiversity, body length, macroecology, Neotropical region, richness

## Abstract

Body size is correlated with many species traits such as morphology, physiology, life history and abundance as well; it is one of the most discussed topics in macroecological studies. The aim of this paper was to analyze the body size distribution of Chrysomelidae, caught with Malaise traps during two years in four areas with different levels of conservation in the Araucaria Forest, Paraná, Brazil, determining if body size is a good predictor of abundance, and if body size could be used to indicate environmental quality. Body size was considered the total length of the specimen from the anterior region of head to the apex of abdomen/elytron. Measurements were taken for up to ten specimens of each species for each area and for all specimens of those species represented by fewer than ten individuals. The highest abundance and richness of Chrysomelidae were obtained in the lowest body size classes. This herbivorous group showed a trend toward a decrease in body size with increasing abundance, but body size was not a good predictor of its abundance. There was a trend toward a decrease in body size from the less to the most conserved areas; however, the definition of a pattern in successional areas not seems to be entirely clear.

## Introduction

Potential ecological relationships between body size and structure of animal communities have been one of main focuses in ecological studies (Braun et al. 2004). Body size is correlated with many morphologic, physiologic, behavioral and ecologic traits, such as dispersal capacity, metabolic and digestive efficiency, reproduction rate, and generation time, as well as species abundance (Siemann et al. 1999, Brown 2003, White et al. 2007). In macroecological studies, the relationship between body size and abundance is one of the most studied topics, and reports mainly concern vertebrates (Krüger and McGavin 2000).

The relationship between body size and abundance is an essential link between individual and population level traits and the structure and dynamics of ecological communities (Woodward et al. 2005). According to White et al. (2007), there are four distinct, but interrelated, relationships between body size and abundance, which are generated by different combinations of processes and routinely confused. The relationships are: i) local size-density relationships which reflect processes influencing resource allocation among species; ii) individual size distributions which result from processes governing the distribution of individual sizes; iii) cross-community scaling relationships which are generated by general constraints, such as resource limitation, on the community as a whole; and iv) global size-density relationships which reflect ecological and evolutionary processes on large spatio-temporal scales.

Controversy has arisen regarding how body size and abundance are related, and concerning the ecological and evolutionary implications of these relationships. In this way, an early step in elucidating the factors that structure animal assemblages may be to understand how the body sizes of their component species are distributed (Blackburn and Gaston 1994, 1997). Hutchinson and MacArthur (1959) suggested that within a taxon there are more species of intermediate size than very large or very small ones, because they would be relatively more specialized and would utilize their resources better, since they would have a larger number of niches available. Following the energetic equivalence rule (EER) proposed by Damuth (1981, 1991) the amount of energy that a population of a species uses in the community is independent of its body size. Damuth (1981) found a slope of -0.75 for the relationship between population density and body size, and since body size scales with metabolic rate to the 0.75 power, the population density would compensate for the body size.

Another aspect about the body size that has been widely discussed is its use in the assessment of environmental quality. In general, richness and abundance are the variables most used to measure not only the diversity but also to assess the environmental quality of areas in different successional stages. Studies have shown that habitat type, management, succession and degradation level have a great influence on the body size of insects increasing or decreasing species body size along succession (Blake et al. 1994, Siemann et al. 1999, Brändle et al. 2000, Braun et al. 2004, Gaucherel et al. 2007). According to Siemann et al. (1999), this variation in size could be related to species efficiency-specialization to different habitats where large and efficient species would be benefited in initial stages of succession while the small and specialized ones would be benefited in final stages of succession. In this way changes in species body size over the succession could be another important indicator of environmental changes and quality.

Phytophages represent about 45% of all described insect species (Frenzel and Brändle 2001). Among them, Chrysomelidae is one of the most diverse groups with more than 36,000 described species (Bouchard et al. 2009) and its body size has never been the focus of study. Thus, the first aim of this paper was to analyze the relationship between body size and abundance and between body size and richness in a Chrysomelidae community, to determine how these variables are related. Two applicable relationships proposed by White et al. (2007) were tested: the individual size distributions, regardless of the identity of species, and the local size-density relationships, since cross-community scaling relationships are more often used in studies of sessile communities and global size-density relationships needs, where data are generally on a wide geographic scale and from a larger number of groups. The second aim was to determine if there are differences in body size between areas at different levels of succession and if so how great.

## Material and methods

The data came from the project Vila Velha (PROVIVE), which was developed in the Parque Estadual of Vila Velha (25°13'5.0"S; 50°2'26.9"W). This park is a conservation unit in the state of Paraná with an area of 3.122 ha, mainly covered by natural fields (steppe, grassy-woody) (Ziller 2000) associated with the Araucaria Forest at different levels of anthropic interference. The park is located in Ponta Grossa at an altitude of 880 m.

Of the five areas sampled during the PROVIVE project, the material from four areas was used in this study, one edge area and three with increasing conservation level. A brief description of these areas is as follows. More information could be found in Ganho and Marinoni (2003): a) Border, an edge area of transition between field and Araucaria Forest in intermediate stage of succession, maintained by mowing; b) Phase 1, area of about 15 ha, previously used for seasonal crops such as corn and beans, in natural regeneration since 1984. It was at an initial to intermediate level of succession; c) Phase 2, primary forest, changed by the removal of various plant species such as *Araucaria angustifolia* (Bert.) O. Ktze (Araucariaceae), *Ocotea porosa* (Nees & C. Mart.) Barroso (Lauraceae) and some Myrtaceae. Plant succession is at an intermediate to advanced stage; d) Phase 3, primary forest changed by selective cutting. It is the best preserved of all, showing a very similar flora to Phase 2 area, but with higher density of araucarias, epiphytes and lianas.

In each sampling area, a Malaise trap was placed and the caught material removed weekly from September 1999 to August 2001. As Malaise is a selective trap collecting flying insects, in this study Chrysomelidae assemblage is composed by species that fly from ground to 2m high and, because of the sampling effort, it was assumed that this trap sampled all species that occur in each sampling area.

The Coleoptera were mounted, labeled, and the chrysomelids identified to the lowest taxonomic level possible. The material is deposited in the Coleção de Entomologia Pe. J. S. Moure, Departamento de Zoologia, Universidade Federal do Paraná (DZUP).

All Chrysomelidae species sampled in each area were measured. The size was considered the total length of the specimen from the anterior region of the head (excluding antennae) to the apex of the abdomen or elytra (Morse et al. 1988). Therefore, the head (superior margin of eyes to the apical margin of labrum, in frontal view), the pronotum (in central region) and elytra/abdomen (sutural margin, in dorsal view) were measured separately. These three measures were summed, resulting in the length of each specimen. Measurements were made with a Wild-M5 stereomicroscope using an adjusted ocular micrometer.

According to Morse et al. (1988), the length of most beetle species varies little; the difference between the largest and the smallest specimen does not exceed 10%. Thus, following the methodology proposed by these authors, measurements were taken from a maximum of ten specimens of each species for each area or all specimens for those species represented by fewer than ten individuals.

The length values were grouped in arbitrarily established size classes (class 1: 1.0 to 2.99 mm, class 2: 3.0 to 4.99 mm, class 3: 5.0 to 6.99 mm and so on) and adjusted on a logarithmic scale, following Morse et al. (1988). This was done for all Chrysomelidae data and for each examined area separately. To determine the distribution of abundance of individuals, the identity of species was not taken into account, so one species may have individuals of more than one size class. The average size of each species was used to treat the distribution of species.

Correlation analyses were performed between size classes and abundance and, between size classes and richness.

A regression analysis was performed to determine the influence of body size on the abundance of Chrysomelidae. The dependent variable was the abundance of each species and the independent variable its average size. The slope obtained was visually compared to that proposed by Damuth (1981). This analysis was performed for each area separately and subsequent for all areas combined.

To examine if there were differences in body size of the Chrysomelidae community in each area, ANOVA (5% significance) was performed based on all measured values. This analysis was also used only for those species recorded in all areas. In addition, ANOVA was used to determine if the size of species varied in areas with different succession levels. For this, species that occurred at least in two areas and that had at least six specimens sampled were selected. Each species was analyzed separately, totaling 15 species that met these prerequisites.

The normality of data was previously tested by the Kolmogorov-Smirnov test and data were log transformed. The analyses were performed using the STATISTICA program 8.0 (StatSoft. Inc. 2007).

## Results

During the two years 2,650 specimens of 254 Chrysomelidae species were sampled and, 1,217 specimens were measured (Table 1).

**Table 1.** Body size (mm) (mean ± SD) of the Chrysomelidae community, trapped with Malaise in four areas with different conservation levels, in Ponta Grossa, Paraná, Brazil. Values followed by the same letter do not differ significantly (*P* < 0.05). (n) number of specimens measured, (S) richness and (N) abundance.

	**Body size**	**n**	**S**	**N**
**Borde**r	6.23 ± 2.42a	391	134	484
**Phase 1**	5.63 ± 2.86b	267	78	742
**Phase 2**	4.75 ± 1.94c	317	88	1010
**Phase 3**	5.38 ± 2.50b	242	70	414
**Total**	-	1,217	254	2,650

**Figure 1.**
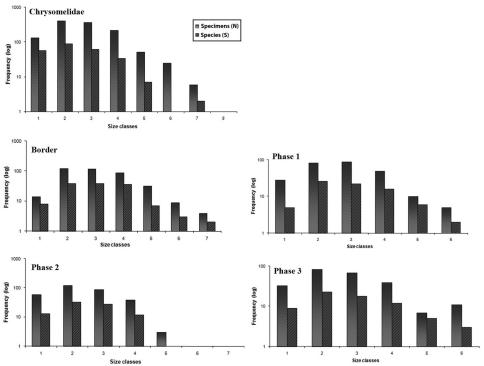
Size Frequency of specimens (N) and species (S) of Chrysomelidae total and in each area, with different conservation levels, in Ponta Grossa, Paraná, Brazil.

The Chrysomelidae size class histogram showed a tendency toward a decrease in abundance with increase in body size (Fig. 1), where the pattern of Chrysomelidae distribution of abundance was a polygonal type with a tail to the right. The same pattern was observed for the richness distribution. The highest frequencies of both abundances and richness were in class 2, with chrysomelids measuring from 3.0 to 4.99 mm.

The distribution of abundance in each area also showed a tendency toward a decrease in abundance with increasing body size, with the highest frequencies in class 2. However, in Phase 1 a higher abundance of Chrysomelidae was in class 3, from 5.0 to 6.99 mm and, in Phase 3, unlike the other, showed the highest abundance in class 6, from 11.0 to 12.99 mm (Fig. 1).

Regarding species richness, this followed the distribution pattern of abundance, with the largest number of species occurring in smaller size classes. Border area had the same number of species in classes 2 and 3. Notice that class 6 to all Chrysomelidae and class 5 of Phase 2 (Fig. 1) there are no values of richness. It happened because there are no species that fit in these size classes, i.e., the average species size fitted in other size class. However, as the specimens have a range of size, some values fitted in different size classes.

In all areas, there was a negative correlation between body size and abundance and between body size and richness, but only in Phase 1 and Phase 2 these correlations were significant (Table 2).

**Table 2.** Correlation between size class and abundance (N) and between size class and richness (S) of Chrysomelidae trapped with Malaise in four areas with different conservation levels, in Ponta Grossa, Paraná, Brazil. Values followed by * showed significant correlation (*P* < 0.05).

**Areas**	**N**	**S**
**Border**	-0,47	-0,49
**Phase 1**	-0,70*	-0,67*
**Phase 2**	-0,67*	-0,64*
**Phase 3**	-0,53	-0,52

**Figure 2.**
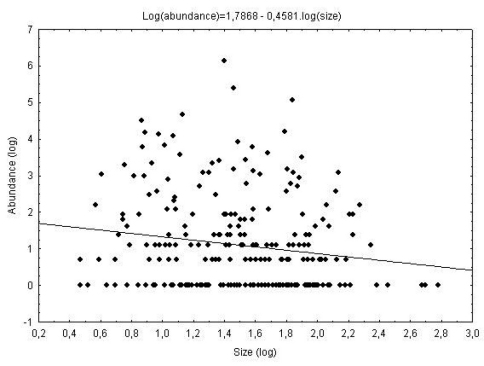
Relation between body size (log) and abundance (log) of Chrysomelidae, trapped with Malaise in four areas with different conservation levels in Ponta Grossa, Paraná, Brazil (closed circles = observed data, line = linear model adjusted).

Studying the influence of body size on abundance, it was possible to show that the model was significant (*b* = -0.46, r = 0.16, *P* < 0.01) only when data from all areas are included (Fig. 2). Even so, body size explained only 2.56% of Chrysomelidae abundance. Moreover, the slope was -0.46.

The Border area, which is an ecotone between a field and Araucaria Forest and which is influenced more by human activity, was the place where the species reached the highest body sizes, 6.23 mm on average, and it was the only area with size class 7, with chrysomelids measuring from 13.0 to 15.0 mm. In this same area was sampled the higher number of species. In contrast, Phase 2 which is an intermediate stage of conservation, showed the smallest size, 4.75 mm on average, with the maximum size occurring in class 5 and, where was registered the higher abundance. The lowest richness as well as the lowest abundance was in Phase 3 (Table 1).

There was a significant decrease (F_3, 1213_ = 28.7, *P* < 0.05) in chrysomelid body size in areas less conserved, Border and Phase 1 to Phase 2 (Table 1). However, in Phase 3, which is the best conserved area, size was significantly greater than that of Phase 2 and did not differ significantly from that of Phase 1. There was no difference in body size of the Chrysomelidae community when only the species common to all areas were analyzed.

While determining if species common to at least two of the studied areas showed variation in body size, it was found that of the 15 species examined, eight had an increase in body size from an area less conserved to one better conserved, but for only two of them, *Trichaltica elegantula* Baly, 1876 and Hispini sp.9, this increase was significant. Four species showed a decrease in body size from an area less conserved to a better conserved, but this difference was not significant in any of the cases. *Acanthonycha costatipennis* Jacoby, 1905 and Eumolpinae sp.1 showed a significant increase in body size from the edge area to an area of intermediate level of conservation, followed by a significant decrease in size in the most conserved area, Phase 3, regarding to compared to the edge (Table 3).

**Table 3.** Body size (mm) (mean ± SD) of Chrysomelidae species common to at least two of the four areas with different levels of conservation and which have at least six specimens collected in Ponta Grossa, Paraná, Brazil. Averages followed by the same letter in line do not differ significantly (*P* < 0.05).

	**Border**	**Phase 1**	**Phase 2**	**Phase 3**
*Acanthonycha chloroptera*		5,19±0,80a	5,65±0,46a	
*Acanthonycha costatipennis*	4,96±0,72**a**		5,84±0,28**b**	5,07±0,59**b**
*Dinaltica gigia*		5,15±0,31a	4,89±0,29a	
*Heikertingerella ferruginea*	3,74±0,36a		3,68±0,21a	3,78±0,16a
*Monoplatus ocularis*	3,92±0,25a	3,82±0,22a	3,72±0,14a	
*Neothona prima*		2,20±0,13a	2,33±0,11a	
*Omophoita octoguttata*	10,11±0,77a	10,41±0,62a		
*Phyllotrupes violaceomaculatus*		7,80±0,52a	7,96±0,64a	
*Trichaltica elegantula*			2,40±0,18**a**	2,65±0,15**b**
Hispini sp.7		6,90±0,44a		7,04±0,41a
Hispini sp.9	5,97±0,30**a**	6,25±0,19**b**		
Eumolpinae sp.1	5,00±0,40**a**	5,80±0,31**b**		5,47±0,25**b**
Eumolpinae sp.6	4,88±0,33a		5,05±0,40a	
Eumolpinae sp.14	8,29±0,52a			8,22±0,53a
Eumolpinae sp.15		7,73±0,49a	7,63±0,55a	7,33±0,58a

## Discussion

The highest richness and abundance was recorded in smaller size classes, with the highest number of species and specimens ranging from 3.0 to 4.99 mm in length (Fig. 1). This value was very near to that found for Chrysomelidae fauna by different authors using different collecting methods and in different habitats. Basset and Samuelson (1996), in studying the arboreal community in Papua New Guinea using several collecting methods, recorded the majority of species ranging from 2.8 to 3.3 mm. Pinheiro et al. (1998) who carried out their study in the Brazilian savanna using nets, found the highest frequencies at the same body size class interval as observed here. These authors also recorded a negative correlation between body size and abundance and between body size and richness for Coleoptera, similar to the results obtained here for the Chrysomelidae.

According to Blackburn and Gaston (1997) there are three different species distribution patterns, linear negative, non-linear negative and polygonal. The Chrysomelidae exhibited a polygonal distribution of abundance. Similar results were obtained by Krüger and McGavin (2000), analyzing a local community of insects collected from *Acacia*. They also found a negative relationship between size and abundance as in this paper.

In fact, according to Blackburn and Gaston (1997), distribution patterns of abundance are strongly influenced by the scale of study. Polygonal relations are usually obtained when unique areas are sampled trying to estimate the abundance of all species of a taxon that occur there, usually using a single and consistent method. In contrast, a negative linear relationship is obtained from compiled data from a great variety of published papers, which generally deal with one or a few species that occupy large geographic areas and whose density is estimated using a wide range of methods. These patterns, according to the authors, are not mutually exclusive and may indicate a clear relationship between abundance and body size at different spatial scales.

According to Morse et al. (1985), an inverse relationship between size and abundance, especially for herbivorous insects, would be linked to the fractal structure of plants. The fractal theory predicts that area or length becomes disproportionately large with a decrease in the unit of measure. Thus, the consequence of the fractal nature of the environment for the species body size distribution occurs due the existence of a more usable space for smaller animals, so species of smaller size should be more represented in nature, as they could subdivide the habitat and coexist in greater numbers.

However, the fractal structure of environment cannot alone explain the shape of the size distribution, since the smallest size class is not always the most numerous, but this may be a mechanism that accounts for the shape of distribution (Kozlowski and Gawelczyk 2002). Although, the distribution pattern of Chrysomelidae abundance is in accordance with those found for other local insect communities, their explanation in terms of ecological processes involved is complex and cannot be summarized in a simple cause and effect relationship.

Size is a poor predictor of Chrysomelidae abundance. Other variables such as availability and quality of food resources, presence of predators/parasitoids, intra- and interspecific competition and climatic factors should have a greater influence on the abundance of this group.

Several authors have found that body size is a poor predictor of population densities on a local scale (Morse et al. 1988, Blackburn et al. 1993, Blackburn and Gaston 1997, White et al. 2007). Furthermore, data obtained here did not support the EER, indicating that larger species use the most available resources. Brown and Maurer (1986) also showed that the greater abundance of small-sized species is not sufficient to compensate for their lower rates of energy use per individual.

It is important to stress that EER as calculated here is not recommended by White et al. (2007). According to these authors, data obtained on a local scale represent a small portion of data needed to test EER, and consequently, only the lower limits of species distribution would be included in the calculations. EER provides global patterns, which are not strictly ecological, but has an important evolutionary component. On the other hand, processes at local scales are more influenced by the partition of resources within the community (Allen et al. 2006, White et al. 2007).

Many features of organisms are correlated with animal body size, but especially life history, ability of dispersion and efficiency, and feeding specialization are linked to succession, so that changes in body size during plant succession may be an important indicator of environmental changes (Siemann et al. 1999). The Chrysomelidae exhibited a trend toward a decrease in body size from Border area, Phase 1, Phase 2 (Table 2); however, the pattern still seems to be unclear, due to the increase in the most conserved area, Phase 3. Besides, in the richest area (Border) the spectrum of body size measurements is very high comparing to the lowest rich area (Phase 3). Consequently, there is a high probability of increasing the body size in the richest area since increasing the spectrum usually increases the average. On the other hand, in Phase 2 where the second richest value was found, the spectrum has no influence since it was found the smallest size. Furthermore, we could not methodologically limit the number of species to eliminate some possible spectrum effect because it would change the composition of the local fauna and, although it seems to have a gradient relating the number of species with body size, the higher number of species alone does not explain the size found.

In the literature, there are different results, some of them show the same tendency as in this study, such as those of Siemann et al. (1999) and Braun et al. (2004). Siemann et al. (1999) studied the dynamic of arthropods in areas with different succession stages and found that, among the analyzed guilds (parasites, predators, herbivores and detritivores), only the herbivores had a significant decrease in body size with increase in age of area. According to these authors, the explanation for decreasing body size of herbivores is the tradeoff between efficiency and specialization.

There are environmental changes that could favor different species at different stages of succession. In early succession, plants have few defenses, high growth rates and low proportion of carbon and nitrogen in their tissues (Tilman 1990). As large animals have greater digestive and absorption efficiency due to larger guts, they may be able to overcome the small herbivores. In later succession, plants are less palatable, have lower growth rates and higher proportion of carbon and nitrogen in their tissues. Since smaller species may perceive greater levels of heterogeneity, small herbivores may be ablest to specialize on certain plants or parts of plants such as growing leaf tips or phloem cells, which have better nutritional quality, prevailing over the large and efficient herbivores (Siemann et al. 1999).

Braun et al. (2004) also found larger body sizes in less conserved areas, even working on a Coleoptera predator group. They studied the Carabidae fauna of areas in regeneration after the closure of a fertilizer factory. According to them, before the factory closure, there were few herbivorous species, which were large and generalists, and thus prey for large Carabidae. With the factory closure, there was a reduction of local pollution, allowing a recovery of vegetation. Thus, with the increase in primary production, there was an increase in immigration of herbivores expanding the food availability for carabids. Furthermore, the authors suggested that habitat structure must have also influenced the Carabidae body size, interfering with foraging efficiency. Early stages of succession and more open areas favored large species which are more efficient at traveling over larger distances in a patchy environment compared to later stages with denser vegetation, where smaller and more agile species may be favored.

Other studies, however, have found opposite results. Blake et al. (1994) reported that both habitat type and management level had a significant influence on Carabidae body size, and concluded that disturbed habitats support a smaller body size fauna. Brändle et al. (2000) observed that in succession areas, there was dominance of macropterous Carabidae species of small size in early stages and brachypterous species of larger size in later stages of succession. Gaucherel et al. (2007), also studying Carabidae, recorded that the intensification of agriculture has a greater impact on large-sized species, so that small species predominate in more disturbed areas.

As these results conflict with those obtained here and mainly do not deal with herbivorous insects but predators, it appears that the efficiency-specialization hypothesis proposed by Siemann et al. (1999) seems to be the most consistent explanation for the decrease in body size of Chrysomelidae in the most conserved areas, at least for this data set.

Among the 15 species examined, only four showed significant variations in body size among the different areas, and consequently, it was not possible to establish a consistent pattern between body size and level of conservation when species were analyzed separately. However, interesting information was obtained. Unlike what was determined for the entire Chrysomelidae community where the edge area had the highest average body size, for three species that showed significant differences in body size among areas (*Acanthonycha costatipennis*, Hispini sp.9 and Eumolpinae sp.1), the Border was the area where these species had the smallest body size. These species did not contribute to explaining the Chrysomelidae pattern, where it was not possible to know which species most influenced the pattern.

## Conclusions

The Chrysomelidae, an essentially phytophagous group, showed a trend toward a decrease in abundance with increasing body size, in a negative polygonal relation. Furthermore, a greater number of chrysomelid species collected by Malaise traps occurred in smaller body size classes; species ranged from 1.0 to 15.0 mm in length and most of them measured between 3.0 and 4.99 mm.

The results presented here seem to follow the pattern found for several animal groups, where body size is a poor predictor of abundance. Other factors such as availability of food, metabolic efficiency, host plant specificity and/or parts of the plant, predation, parasitism and climate should act more on the Chrysomelidae community determining the size of species populations.

It was demonstrated that there is a change in body size of Chrysomelidae communities in areas with different levels of conservation. There was a trend toward a decrease in body size of the less to the most conserved areas. The Border area, which is an ecotone and more influenced by human activity, had larger chrysomelid body sizes. However, the definition of a pattern in successional areas did not seem to be entirely clear, due to significant increase in body size of a later succession stage in relation to one of the others areas in an intermediate successional stage. Nevertheless, the results suggest that degrading the habitats, the small and specialized species would be at risk of disappearing.

The fractal characteristic of the environments, mainly the plants, may be one of the operating mechanisms in Chrysomelidae community. It would explain the higher richness and abundance of this group into smaller size classes, but it should not be considered the only explanation. Other factors, such as those already mentioned could be interfering in the ecological processes that generate such patterns.
